# The Training of Pauper Girls

**Published:** 1905-07-01

**Authors:** 


### THE TRAINING OF PAUPER GIRLS.

The Guardians of the West Derby Union may not, unfortunately, be a peculiar people, but they have but a limited notion of their duties, if we may judge from the report of a discussion held some months ago among them regarding the training of girls. Some of the Guardians seem to think that it was mere folly to train the girls under their care for domestic service, and declared that the people who took these girls were only "second-hand paupers" themselves. It is rather difficult to say what is meant by a "second-hand pauper," but we presume that the West Derby Guardians meant a person who could not afford to be fastidious. It did not seem to strike those who objected to spending money on the training of the girls that the result might be to place them in the houses of something better than "second-hand paupers." In the present dearth of domestic servants, there is an abundance of opportunity to place out girls from Poor-law schools in good houses, if only they have been properly trained. In some of the metropolitan Poor-law schools, the matrons receive more requests for girls as servants than they can comply with, and are careful to place their graduates only in situations of which they know something good; when the girls leave the school they are generally put in the care of either the Metropolitan Association for Befriending Young Servants or the Girls' Friendly Society, so that some kindly care and supervision of them is exercised during those first years of liberty in which they are most apt to take up with undesirable companions, and otherwise get into danger. Girls from well-managed Poor-law schools are particularly well-suited for subordinate posts in large households. They have been well-trained in punctuality, cleanliness, and obedience, and therefore get on well with upper servants. They are more often lacking in initiative and in making shift without all possible contrivances and utensils—unless in so far as their memory of pre-school life may come in—and therefore may be less adapted for service in modest households, and are peculiarly unfitted for service with "second-hand paupers" whose life is one continual makeshift. This lack of invention and flexibility may perhaps tell against them if they marry without having gained any knowledge of making the best of limited means, but this, after all, depends more on the native capacity of the girl than on her training. And the main thing for guardians to remember is that by the proper training of the girls under their care they may put them in the way of earning a good and honest living, which should raise them permanently above pauperism.

